# Matching for HLA-DR excluding diabetogenic HLA-DR3 and HLA-DR4 predicts insulin independence after pancreatic islet transplantation

**DOI:** 10.3389/fimmu.2023.1110544

**Published:** 2023-03-21

**Authors:** Cassandra Ballou, Franca Barton, Elizabeth H. Payne, Thierry Berney, Jean Villard, Raphael P. H. Meier, David Baidal, Rodolfo Alejandro, Mark Robien, Thomas L. Eggerman, Malek Kamoun, Yannick D. Muller

**Affiliations:** ^1^ Collaborative Islet Transplant Registry Coordinating Center, The EMMES Company, LLC, Rockville, MD, United States; ^2^ Division of Transplantation, Department of Surgery, Geneva University Hospitals, Geneva, Switzerland; ^3^ Department of Genetic, Laboratory and Pathology Medicine, Geneva University Hospitals, Geneva, Switzerland; ^4^ Department of Surgery, School of Medicine, University of Maryland, Baltimore, MD, United States; ^5^ Department of Medicine and the Diabetes Research Institute, University of Miami Miller School of Medicine, Miami, FL, United States; ^6^ National Institute of Allergy and Infectious Diseases, National Institutes of Health, Bethesda, MD, United States; ^7^ National Institute of Diabetes and Digestive and Kidney Diseases, National Institutes of Health, Bethesda, MD, United States; ^8^ Immunology and Histocompatibility Testing Laboratory, University of Pennsylvania Perelman School of Medicine, Philadelphia, PA, United States; ^9^ Division of Immunology and Allergy, Centre Hospitalier Universitaire Vaudois and University of Lausanne, Lausanne, Switzerland

**Keywords:** islet transplantation, HLA-DR4, HLA-DR3, insulin independence, type 1 diabetes, insulin, HLA, Human leukocyte antigen

## Abstract

**Introduction:**

In pancreatic islet transplantation, the exact contribution of human leukocyte antigen (HLA) matching to graft survival remains unclear. Islets may be exposed to allogenic rejection but also the recurrence of type 1 diabetes (T1D). We evaluated the HLA-DR matching, including the impact of diabetogenic HLA-DR3 or HLA-DR4 matches.

**Methods:**

We retrospectively examined the HLA profile in 965 transplant recipients and 2327 islet donors. The study population was obtained from patients enrolled in the Collaborative Islet Transplant Registry. We then identified 87 recipients who received a single-islet infusion. Islet-kidney recipients, 2nd islet infusion, and patients with missing data were excluded from the analysis (n=878).

**Results:**

HLA-DR3 and HLA-DR4 were present in 29.7% and 32.6% of T1D recipients and 11.6% and 15.8% of the donors, respectively. We identified 52 T1D islet recipients mismatched for HLA-DR (group A), 11 with 1 or 2 HLA-DR-matches but excluding HLA-DR3 and HLA- DR4 (group B), and 24 matched for HLA-DR3 or HLA-DR4 (group C). Insulin-independence was maintained in a significantly higher percentage of group B recipients from year one through five post-transplantation (p<0.01). At five-year post-transplantation, 78% of group B was insulin-independent compared to 24% (group A) and 35% (group C). Insulin-independence correlated with significantly better glycemic control (HbA1c <7%), fasting blood glucose, and reduced severe hypoglycemic events. Matching HLA-A-B-DR (≥3) independently of HLA- DR3 or HLA-DR4 matching did not improve graft survival.

**Conclusion:**

This study suggests that matching HLA-DR but excluding the diabetogenic HLA-DR3 and/or 4 is a significant predictor for long-term islet survival.

## Introduction

Human Leukocyte Antigens (HLA), also named the major histocompatibility system, represent the main immunological barrier in allogenic transplantation involving direct and indirect antigen recognition pathways (1, 2). Since the first successful kidney transplant between identical twins in the 1950s, it is well accepted that HLA matching for class I (HLA-A, HLA-B) and class II (HLA-DR, HLA-DQ) significantly reduce the risk of acute and chronic rejection, decrease the risk of allosensitization, improve graft function and increase patient survival (3–5). Thus, data from the collaborative transplant study and others demonstrated a stepwise increase in allogeneic kidney graft survival at five-year post-transplantation when increasing the degree of HLA matching (https://www.ctstransplant.org) (6, 7).

On the other hand, HLA antigens are also essential contributors to autoimmune diseases’ pathogenesis. In type 1 diabetes (T1D), specific allele combinations have been associated with >40 increased odds ratio of developing this disease. These include the DRB1*0301-DQA1*0501-DQB1*0201 (DQ2.5) and the DRB1*0401-DQA1*0301-DQB1*0302 (DQ8) haplotypes (8). In pancreatic islet transplantation, the exact contribution of HLA matching on graft survival remains unclear. The frequent need for multiple islet donors for one recipient, the numerous confounding factors influencing successful islet isolation and transplantation, and the overall low number of islet transplants performed worldwide hampered those analysis (9–11). Additionally, despite sustained and measurable improvement of glycemic control and a significant reduction in severe hypoglycemia episodes, long-term (>10year) insulin independence remains only exceptional after islet transplantation (12–16).

This study retrospectively analyzed donor and T1D recipient’s HLA profile as reported in the Collaborative Islet Transplant Registry (CITR), the most comprehensive human-to-human islet transplant data collection. We analyzed the impact of HLA-DR matching including the diabetogenic HLA-DR3 and HLA-DR4 on long-term insulin-independence, fasting C-peptide levels, HbA1c %, and severe hypoglycemic events.

## Methods

### Patients

The CITR is a registry including pancreatic allogeneic islet transplant of Phase I/II/III clinical trials and standard of care since 1999 from North America, European and Australian centers. The CITR is supported by the National Institute of Diabetes and Digestive and Kidney Diseases (NIDDK) and the Juvenile Diabetes Research Foundation International (2006-2015). T1D islet recipient’s data were previously described (17) and are publicly available in CITR Annual Reports (https://citregistry.org/content/citr-11th-annual-report). The Data Coordinating Center verified participant voluntary informed consent.

Briefly, T1D islet recipients are aged between 18- and 65-year-old and have T1D for at least five years with a documented negative fasting C-peptide (<0.3ng/mL). Additionally, they have impaired hypoglycemia awareness and suffer from repetitive and severe hypoglycemia episodes reflected by glycemic lability and elevated HbA1c levels (>8%). In the first part of the study, HLA antigens (A, B, DR) were assessed in 965 islet transplant recipients (including islet alone, islets after kidney, simultaneous islet kidney, and kidney after islet) and 2327 pancreas donors enrolled from 1999 until 2019. The HLA-matching analysis was then conducted in T1D recipients with single islet infusion (N=87). The outcomes were computed in follow-up post infusion for up to five years or until the next infusion or last available follow-up. One year of follow-up was minimally required. Seven recipients received an islet infusion pooled from two donors. Fasting C-peptide, insulin independence, HbA1c levels and severe hypoglycemic events were analyzed. We defined 5 groups: (A) zero HLA-DR matching; (B) 1 or 2 HLA-DR matching, but excluding HLA-DR3-DR4 matching; (C) HLA-DR3 and/or DR4 matching; (D) <3 HLA-A, -B, -DR matching; (E) 3-6 HLA-A, -B, -DR matching. Of note, Group B could include HLA-DR3 or HLA-DR4 donors if they were mismatched to the recipients (n=2/11).

### Classification of human leucocyte antigens

From a total of 1403 allograft islet recipients, 965 had HLA data entered in the CITR. Data on HLA class I (A, B) specificities and class II (DR) specificities in islet donors (N=2327) were also retrieved from the CITR registry. This analysis was performed independently of the exclusion criteria used to study the role of HLA-DR matching (see below). HLAs were defined as present or missing information. The classification of HLA changed over time from serological typing to DNA genotyping. As the precision level of HLA typing varied across techniques (serological versus molecular), time and centers, we regrouped the HLA antigen analysis at the broad and not split level. Split HLA specificities were at first technically undistinguishable at the broad level. As HLAs were reported in the CITR registry either at the broad or split level, we converted split HLA at their broad level to keep the analysis homogenous. Split and broad HLA classification can be found online (http://hla.alleles.org/antigens/broads_splits.html). Because each individual has two alleles, one HLA can be present one or two times. If only one class I or class II HLA was reported, the individual was considered homozygous for this HLA. T1D islet recipients with missing HLA-DR typing information were excluded from the analysis (n=762, 54% of the full database). HLA A/B/DR matches were defined at the broad level. Finally, HLA-C, HLA-DP and HLA-DQ typing were not examined because this information was not collected.

### Statistics

CITR primary efficacy data are collected at protocol-designated time points through 10 years from last transplant, regardless of current or previous total islet graft failure. In the event of complete graft failure and no data collected at the visit, the following data were imputed: insulin use was set as “using exogenous insulin”, and C-peptide was set as 0.0 ng/L. HbA1c, fasting glucose, and SHEs were set to missing and analyzed as missing at random (outcome not related to absence of data).

Multivariate modeling for each primary efficacy outcome (including insulin independence, basal C-peptide, absence of severe hypoglycemic events, and HbA1c) was done *via* mixed effects models with repeated measures per subject (multiple annual visits per patient). Primary efficacy outcomes were analyzed following first infusion.

The prevalence of insulin independence is the optimal way to characterize the probability of being insulin independent in follow-up time post islet transplantation Thus, insulin independence can be lost and re-gained, often over periods spanning months or years. Prevalence also reconciles disparities in factors that may predict retention but not achievement or vice versa.

All reported p-values are observed, nominal, and outside of any pre-planned Type I error structure. In drawing conclusions, readers should be mindful that the significance levels control for random variance, but not systematic biases in the data nor multiple testing. However, these analyses do provide insight and direction for future questions and analyses.

## Results

From 1403 allograft recipients enrolled in CITR, 965 had HLA data entered. The analysis of the HLA-A, -B, -DR broad antigens was conducted in 965 pancreatic islet graft recipients and 2327 donors from the CITR. On the broad antigen level, fold change difference for HLA-A (1, 2, 3, 9, 10, 11, 19, 28, 36 80) expression comparing T1D islet recipients and pancreas donors ranged between 0.5 (HLA-A11) to 1.4 (HLA-A1); for HLA-B (5, 7, 8, 12, 13, 14, 15, 16, 17, 18, 21, 22, 27, 35, 37, 40, 41, 42, 46, 47, 48, 53, 59, 67, 70, 73, 78, 81, 82) between 0.4 (HLA-B37, HLA-B17) to 2.2 (HLA-B8); for HLA-DR (1, 2, 3, 4, 5, 6, 7, 8, 9, 10) between 0.1 (HLA-DR10) to 2.6 (HLA-DR3). Among all, the highest fold change differences were HLA-B8, HLA-DR3 and HLA-DR4, i.e 2.2, 2.6 and 2.1 times more frequent in T1D recipients respectively (**Table 1**).

**Table 1:** Broad HLA specificities in pancreas donor and islet recipients.

Broad Antigen HLA-A	Split Antigens	Donor Frequency (%)	Recipient Frequency (%)	Fold change
1		634 (13.8)	366 (19.7)	1.4
2		1298 (28.2)	596 (32)	1.1
3		682 (14.8)	219 (11.8)	0.8
9	23;24	541 (11.8)	239 (12.8)	1.1
10	25;26;34;66	245 (5.3)	86 (4.6)	0.9
11		289 (6.3)	60 (3.2)	0.5
19	29;30;31;32;33;74	668 (14.5)	237 (12.7)	0.9
28	68;69	224 (4.9)	59 (3.2)	0.7
36		11 (0.2)	0	0.0
80		3 (0.1)	0	0.0
Broad Antigen HLA-B				
5	51;52	315 (6.9)	66 (3.5)	0.5
7		575 (12.5)	132 (7)	0.6
8		438 (9.5)	401 (21.3)	2.2
12	44;45	612 (13.3)	198 (10.5)	0.8
13		104 (2.3)	40 (2.1)	0.9
14	64;65	151 (3.3)	46 (2.4)	0.7
15	62;63	321 (7)	207 (11)	1.6
16	38;39	208 (4.5)	102 (5.4)	1.2
17	57; 58	210 (4.6)	33 (1.8)	0.4
18		237 (5.2)	158 (8.4)	1.6
21	49; 50	129 (2.8)	85 (4.5)	1.6
22	54; 55	108 (2.4)	26 (1.4)	0.6
27		189 (4.1)	57 (3)	0.7
35		440 (9.6)	109 (5.8)	0.6
37		55 (1.2)	10 (0.5)	0.4
40	60; 61	300 (6.5)	145 (7.7)	1.2
41		36 (0.8)	24 (1.3)	1.6
42		21 (0.5)	0	0.0
46		7 (0.2)	0	0.0
47		17 (0.4)	8 (0.4)	1.0
48		15 (0.3)	2 (0.1)	0.3
53		48 (1)	15 (0.8)	0.8
59		1	0	0.0
67		4 (0.1)	2 (0.1)	1.0
70	71; 72	44 (1)	8 (0.4)	0.4
73		1	5 (0.3)	15
78		2	0	0.0
81		5 (0.1)	0	0.0
82		1	0	0.0
Broad Antigen HLA-DR	Split Antigens	Donor Frequency	Recipient Frequency	Fold change
1		462 (10.3)	136 (9.6)	0.9
2	15;16	659 (14.7)	81 (5.7)	0.4
3	17;18	518 (11.6)	422 (29.7)	2.6
4		706 (15.8)	464 (32.6)	2.1
5	11;12	566 (12.7)	64 (4.5)	0.4
6	13;14	687 (15.4)	94 (6.6)	0.4
7		570 (12.8)	78 (5.5)	0.4
8		182 (4.1)	66 (4.6)	1.1
9		75 (1.7)	15 (1.1)	0.6
10		43 (1)	2 (0.1)	0.1

**Figure 1:**
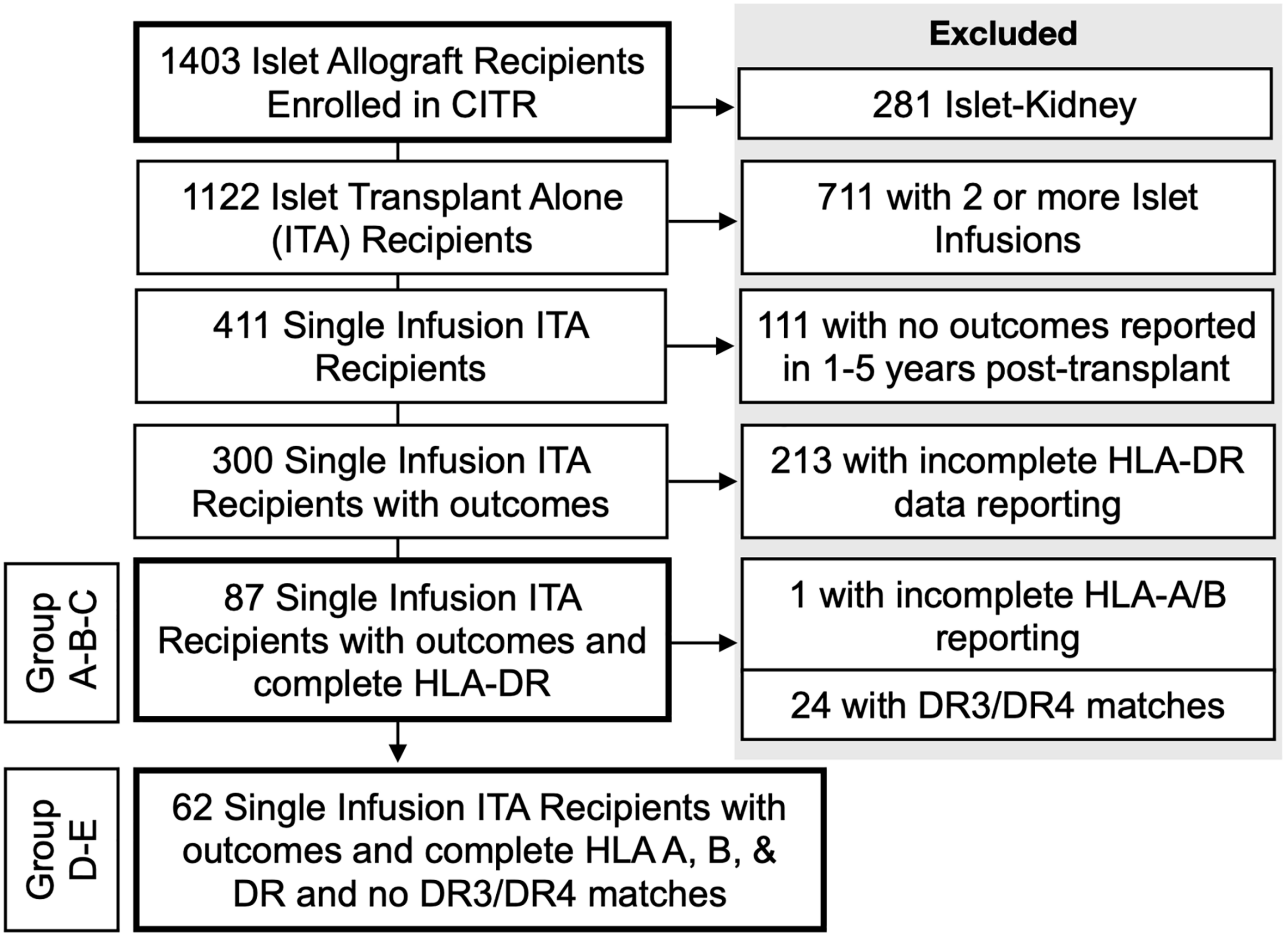
Flow diagram of the islet recipients included in this study.

Since HLA-B8 is often linked with HLA-DR3 representing a conserved haplotype and is not considered *per se* as a risk factor for developing T1D (8, 18–20), we analyzed the contribution of HLA-DR3 or HLA-DR4 matching only. Of 1403 islet recipients, we excluded 281 islet-kidney recipients (islets after kidney, simultaneous islet kidney, and kidney after islet), 711 recipients who received a 2^nd^ islet infusion, 111 recipients with no outcomes reported at years 1-5 post-transplant, and 213 recipients with incomplete HLA-DR data reporting (**Figure 1**). Eighty-seven single-islet transplant recipients were analyzed for this study. Of note, recipients receiving two islet transplants at the same time were not excluded from the analysis. Group A (=high-risk of allogenic rejection) included 52 T1D islet recipients with two HLA-DR mismatches independently of HLA-A and HLA-B matching. Group B (=low risk of allogenic rejection, low risk for autoimmunity) included 11 T1D islet recipients with one or two HLA DR matches, but mismatched for HLA-DR3 and HLA-DR4 (independently of HLA-A and HLA-B matching). Group C (low risk of allogenic rejection, high risk for autoimmunity) included 24 T1D islet recipients with HLA-DR3 and/or HLA-DR4 matches (independently of HLA-A and HLA-B matching). Eight patients were HLA-DR3 positive only, 17 HLA-DR4 positive only and 14 were HLA-DR3 and HLA-DR4 double positive. Key baseline characteristics are described in **Table 2**. Albeit limited by the number of included T1D islet recipients, we noted that the HLA-A and HLA-B matches were similar between group B and C and lower in group A. The number of infused islets equivalent (IEQ), individuals transplanted after 2010, cold ischemia time, cause of donor death, gender, and age were similar between the three groups (**Table 3**). Notably, Group A had significantly fewer recipients than Group B infused with two donors, *i.e* 2 out of 52 compared to 3 out of 11, but not compared to Group C (2 out 24) (**Table 3**).

**Table 2:** Key baseline characteristics of islet transplant recipients and donors.

Mean (S.D.) or %	(Group A)	(Group B)	(Group C)
Recipients	N = 52	N = 11	N = 24
Transplanted after 2010	25%	18%	25%
% Female	69%	64%	63%
Age at Baseline (yrs)	46 (12)	41 (7)	46 (11)
Experiencing SHE at Baseline	88%	73%	91%
Induction Immunosuppression with TCD and/or TNF	8%	0%	8%
Maintenance Immunosuppression with mTOR & CNI	14%	90%	67%
IEQs Infused (1,000s)	482 (188)	583 (159)	495 (202)
Simultaneous islet TX (n, (%))	2 (3.8)	3 (27.3)	2 (8.3)
Genotyping (%Serological)	72%	83%	78%
% with ≥1 Avg. A Matches	48%	73%	63%
% with ≥1 Avg. B Matches	17%	45%	38%
Donors	N = 54	N = 14	N = 26
Age (yrs)	39 (13)	35 (11)	41 (15)
Gender (%Male)	49%	64%	77%
BMI	30.7 (6.2)	33.8 (6.6)	35.0 (8.2)
Cold Ischemia Time (hrs)	8.3 (6.4)	7.0 (2.7)	7.3 (4.0)
Cause of Death: Head Trauma	46%	43%	46%
Cause of Death: Cerebrovascular/Stroke	44%	43%	46%
Cause of Death: Other	10%	14%	8%
Genotyping (%Serological)	60%	57%	52%

Group A, <1 Avg. HLA-DR Matches ITA Recipients with complete HLA & Follow-up data at 1yr post Tx 1.

Group B, ≥1 Avg. HLA-DR Matches (excluding HLA-DR3/4) ITA Recipients with complete DR & Follow-up data at 1yr post Tx 1.

Group C, HLA-DR3 or DR4 Match ITA Recipients with complete DR & Follow-up data at 1yr post Tx 1.

ITA, islet transplant alone; Tx, transplantation; yrs, years; hrs, hours.

**Table 3:** Potential confounders.

P-value	Group B vs. A	Group B vs. C
Transplanted after 2010	>0.9	>0.9
Age at Baseline (yrs)	0.2	0.1
Experiencing SHE at Baseline	0.3	0.3
Baseline Daily Insulin Use (Units/kg) <0.5 UI/insulin/kg	0.7	0.2
PRA I Positive	0.4	>0.9
PRA II Positive	0.7	>0.9
Induction Immunosuppression with TCD and/or aTNF	>0.9	>0.9
Maintenance Immunosuppression with mTOR & CNI	0.2	0.2
IEQs Infused (1,000s)	0.1	0.2
Genotyping (%Serological)	>0.9	>0.9
1 vs. 2 Donors	0.03	0.3
Donor Age <45 years	>0.9	0.2
Gender (%Male)	0.6	0.6
BMI	0.2	0.9
Cold Ischemia Time (hrs)	0.6	0.8
Cause of Death: Head Trauma	0.7	0.5
Cause of Death: Cerebrovascular/Stroke	0.7	0.7
Cause of Death: Other	>0.9	>0.9
Genotyping (%Serological)	0.9	>0.9

Group A, <1 Avg. HLA-DR Matches ITA Recipients with complete HLA & Follow-up data at 1yr post Tx 1.

Group B, ≥1 Avg. HLA-DR Matches (excluding HLA-DR3/4) ITA Recipients with complete DR & Follow-up data at 1yr post Tx 1.

Group C, HLA-DR3 or DR4 Match ITA Recipients with complete DR & Follow-up data at 1yr post Tx 1.

SHE, severe hypoglycemic episodes; PRA, Panel reactives antibody; TCD, T cell depleting reagents; aTNF, anti-TNF antibodies; CNI, calcineurin inhibitors; IEQ, islet equivalent.

**Figure 2:**
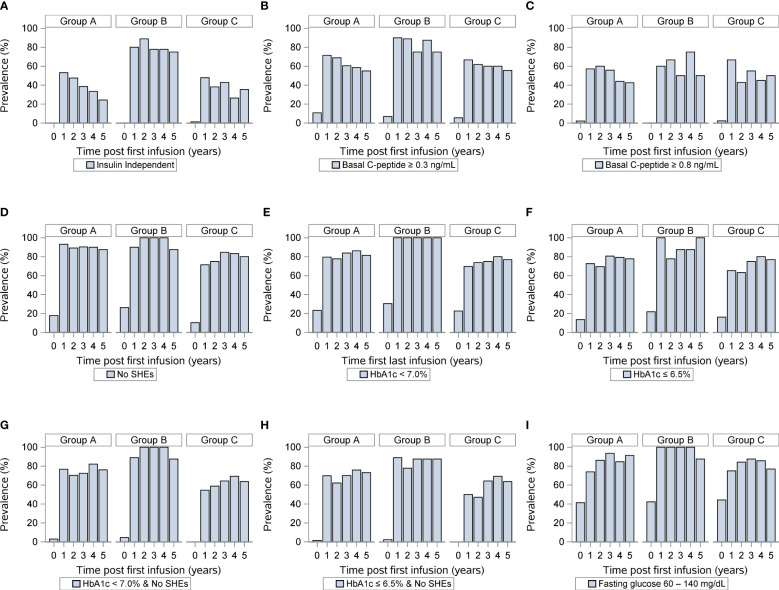
Outcomes of islet transplantation alone with complete HLA-DR data. Insulin independence **(A)**, basal C-peptide ≥ 0.3 ng/mL **(B)**, basal C-peptide ≥ 0.8 ng/mL **(C)**, absence of severe hypoglycemic events **(D)**, HbA1c <7% **(E)**, HbA1c ≤ 6.5% **(F)**, absence of severe hypoglycemic events and HbA1c < 7.0% **(G)**, absence of severe hypoglycemic events and HbA1c < 6.5% **(H)**, fasting blood glucose 60-140 (mg/dL) **(I)** in group A (HLA-DR mismatches) group B (one or two HLA DR matches, excluding HLA-DR3 and HLA-DR4) and group C (HLA-DR3 or HLA-DR4 match). For each panel, the first row indicates years after islet transplantation; the second the percentage with outcome; the third the number of individuals.

We next compared islet transplant outcomes in groups A, B and, C. One-year post transplantation, 53% of the T1D islet recipients reached insulin-independence in group A compared to 82% in group B and 48% in group C. At five-year post-transplantation, the rate of insulin-independence decreased to 24%, 78%, and 35% in group A-B-C respectively (**Figure 2A** and **Table 4**). Concordantly, fasting basal C-peptide level (≥0.3ng/ml), a measure for the durability of islet graft function, was detectable in 71%, 91%, and 67% at 1-year post-transplantation and 55%, 78%, and 56% of individuals at 5-year post-transplantation in group A-B-C respectively (**Figure 2B**). When defining a higher threshold for fasting basal C-peptide (≥0.8ng/ml), group A-B-C had comparable values at 5-year post transplantation (respectively 43%, 56% and 50%, **Figure 2C**). Severe hypoglycemic episodes were mainly absent in all three groups 5-year post-transplantation (**Figure 2D**). Additionally, glycemic control (% HbA1c <7 or HbA1c ≤ 6.5%) was significantly better in group B (**Figures 2E, F** respectively, **Table 4**). The combination for the absence of severe hypoglycemic events and HbA1c <7% or ≤ 6.5 was also significantly better in group B (**Figures 2G, H** and **Table 4**). Finally, fasting blood glucose value (between 60 and 140 mg/dl) was also significantly better in group B (**Figure 2I** and **Table 4**).

**Table 4:** Best predictors by p-value for ITA’s with follow-up data at least 1 year.

P-value	Group A vs B	Group D vs E	Group B vs C
Insulin Independence	0.001	0.8	0.008
Basal C-peptide ≥ 0.3 ng/mL	0.06	0.4	0.1
Absence of Severe Hypoglycemic Events	0.2	0.6	0.07
HbA1c < 7.0%	0.0006	0.0006	0.02
Absence of Severe Hypoglycemic Events and HbA1c < 7.0%	0.003	0.01	0.01
Fasting Blood Glucose 60-140 (mg/dL)	0.004	0.05	0.02

Group A, <1 Avg. HLA-DR Matches ITA Recipients with complete HLA & Follow-up data at 1yr post Tx 1.

Group B, ≥1 Avg. HLA-DR Matches (excluding HLA-DR3/4) ITA Recipients with complete DR & Follow-up data at 1yr post Tx 1.

Group C, HLA-DR3 or DR4 Match ITA Recipients with complete DR & Follow-up data at 1yr post Tx 1.

Group D, <3 Avg. HLA-A, B, & DR Matches ITA Recipients with complete HLA & Follow-up data at 1yr post Tx 1.

Group E, ≥3 Avg. HLA-A, B, & DR Matches ITA Recipients with complete DR & Follow-up data at 1yr post Tx 1.

**Figure 3:**
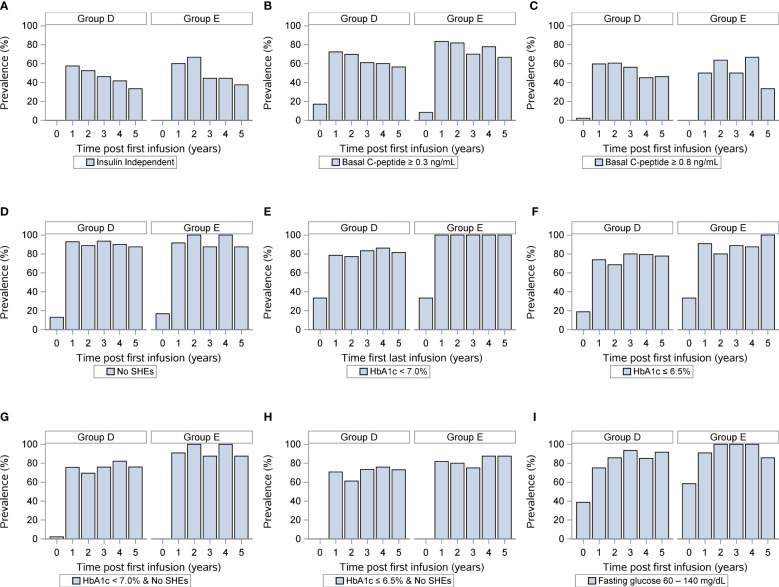
Outcomes of islet transplantation alone with complete HLA-A-B-DR data. Insulin independence **(A)**, basal C-peptide ≥ 0.3 ng/mL **(B)**, basal C-peptide ≥ 0.8 ng/mL **(C)**, absence of severe hypoglycemic events **(D)**, HbA1c <7% **(E)**, HbA1c ≤ 6.5% **(F)**, absence of severe hypoglycemic events and HbA1c < 7.0% **(G)**, absence of severe hypoglycemic events and HbA1c < 6.5% **(H)**, fasting blood glucose 60-140 (mg/dL) **(I)** in group D (<3 HLA A, B, DR matches) and group E (3-6 HLA matches). For each panel, the first row indicates years after islet transplantation; the second the percentage with outcome; the third the number of individuals. P value comparing <3 Avg. Matches vs. ≥ 3 Avg. Matches.

Finally, we compared islet transplant outcomes when only looking at HLA-A, HLA-B, and HLA-DR matching excluding diabetogenic HLA-DR3 and HLA-DR4 matches. We defined two groups, one with less than 3 HLA matching (n=50, group D) and one with 3-6 HLA matching (N=12, group E). Of note 25/87 recipients were excluded, one because of incomplete HLA (A or B) reporting, the other 24 were the DR3/4 matches. Key baseline characteristics of islet transplant recipients and donors were similar between both groups (**Table 5**). Yet, ≥3 HLA matching did not predict long-term insulin-independence, fasting basal C-peptide level, severe hypoglycemic events HbA1c (≤ 6.5%). Only, HbA1c (<7%) and the combination for the absence of severe hypoglycemic events and HbA1c <7% were significantly better in group E (**Figures 3A–I**). Thus, the best predictor for long-term insulin independence was HLA-DR matching, but not for the diabetogenic HLA-DR3 and HLA-DR4.

**Table 5:** Key baseline characteristics of islet transplant recipients and donors.

Mean (S.D.) or %	(Group D)	(Group E)	p-value (D vs. E)
Recipients	N = 50	N = 12	
Transplanted after 2010	24%	17%	0.7
% female	69%	67%	>0.9
Age at Baseline (yrs)	44 (11)	46 (10)	0.7
Experiencing SHE at Baseline	87%	83%	0.7
Induction Immunosuppression with TCD and/or aTNF	7%	9%	>0.9
Maintenance Immunosuppression with mTOR & CNI	68%	81%	0.5
IEQs Infused (1,000s)	497 (194)	498 (158)	>0.9
Simultaneous islet TX (n, (%))	4 (8%)	0	0.6
Genotyping (%Serological)	73%	71%	>0.9
Donors	N = 54	N = 12	
Age (yrs)	38 (14)	41 (9)	0.7
Gender (%Male)	51%	50%	>0.9
BMI	30.3 (5.3)	37.6 (9.3)	0.08
Cold Ischemia Time (hrs)	8.0 (6.5)	7.4 (2.7)	0.8
Cause of Death: Head Trauma	46%	33%	0.5
Cause of Death: Cerebrovascular/Stroke	42%	58%	0.5
Cause of Death: Other	13%	8%	>0.9
Genotyping (%Serological)	63%	55%	0.6

Group D, <3 Avg. HLA-A, B, & DR Matches ITA Recipients with complete HLA & Follow-up data at 1yr post Tx 1.

Group E, ≥3 Avg. HLA-A, B, & DR Matches ITA Recipients with complete DR & Follow-up data at 1yr post Tx 1.

SHE, severe hypoglycemic episodes; PRA, Panel reactives antibody; TCD, T cell depleting reagents; aTNF, anti-TNF antibodies; CNI, calcineurin inhibitors; IEQ, islet equivalent.

## Discussion

This study investigated first the benefit of HLA-DR matching in islet transplantation alone.

We not only confirmed that HLA-DR3 and HLA-DR4 prevalence was higher in T1D islet recipients than donors, but also found that long-term insulin independence was significantly better when matching donor islets for HLA-DR but not the diabetogenic HLA-DR3 and HLA-DR4. This correlated with C-peptide production, better glycemic control and reduced severe hypoglycemia.

Opportunities for HLA matching in islet transplantation have always been limited and therefore poorly studied given the small numbers of patients on the waiting list and the frequent need for multiple islet infusions expositing patients to multiple HLA mismatches. Interestingly, when reviewing data from pancreas transplantation, results are controversial (21). Data published in 2002 by the United Network for Organ Sharing (UNOS) retrospectively analyzing >3000 pancreas transplants performed between 1988-1994 showed no benefit of HLA matching (22). More recently, in pancreas after kidney transplant recipients, 5-6 HLAs mismatches resulted in accelerated time to rejection albeit without any impact on pancreas overall survival and rejection rate (23). A retrospective review of a prospectively assembled database of 1219 pancreas transplants performed at the University of Minnesota found increased rejection rates in HLA-B and HLA-DR mismatched donor-recipient pairs but not HLA-A, -C and HLA-DQ (24). It must be acknowledged that these studies have not specifically examined the impact of HLA-DR3 and HLA-DR4 matching. Other confounding factors such as pre-transplantation HLA sensitization, surgical complications, the immunosuppressive regimen could also have interfered in the interpretation of these results. In our study, the benefit of ≥3 HLA matching was not obvious. Considering that donor selection criteria for islet transplantation are more stringent than for other types of transplant and that there is a significant risk for manufacturing failure, this suggests that HLA-A, -B, -DR matching is hardly feasible in clinical practice.

Evidence from animal studies suggests that autoantigen presentation is more effective in an MHC matched context increasing the risk of T1D relapse (25, 26). Such recurrences are yet difficult to diagnose after islet transplantation (27, 28). As such histological monitoring after intraportal islet infusion is limited, considering the low islet sampling rate  (29). An indirect approach is to monitor autoantibodies against insulin, the insulinoma-associated tyrosine phosphatase- like protein (IA-2), the 65-kDa glutamic acid decarboxylase isoform (GAD65), and zinc transporter 8 (ZnT8) (30–33). In a single case report, a 56-year-old man with a 44-year history of T1D developed hyperglycemia associated with rapidly increasing anti-GAD65 titer 33 months post-transplantation (34). Altogether, these results confirm that islet transplant recipients can develop relapsing T1D.

T1D relapse is more studied after pancreas transplantation. In a prospective cohort of 223 recipients of simultaneous pancreas–kidney allografts, the group of Pugliese estimated that recurrence of autoimmunity occurred in 7% of the cases (mean follow-up was 6.2 years, range 1.0–22.6). A selective loss of beta cells was proven histologically (35). Identified risk factor for relapsing T1D were post-transplant autoantibody positivity, autoantibody number, and autoantibody conversion. Importantly, the authors found that the DR3/DR4 genotype increased the risk of islet autoantibody seroconversion and thereby the risk of T1D relapse (35). A recent multicentric prospective cohort monitoring 24662 children at increased risk for T1D, further confirmed that autoantibodies conversion, from single to multiple, and the HLA DR-DQ (DR4-DQ8/DR3-DQ2.5) haplotype predicted the risk of progression to T1D (32). These results support the hypotheses that the diabetogenic HLA-DR3 and HLA-DR4 trigger disease relapse after pancreatic and islet transplantation.

This study has several important limitations. First, only a limited number of T1D islet recipients could be included hampering more extensive analysis on the mechanisms driving the loss of insulin-independence and the potential confounding factors. Future sub-analyses comparing outcomes of HLA-DR3 versus HLA-DR4 or HLA-DR3/DR4 double positive (n=14) T1D patients are warranted. Additionally, some protective loci, such as HLA DR15 (DR2 in the old nomenclature) for the development of type I diabetes could represent a significant confounder in our analysis (36). In future studies, islet loss of function should be correlated with autoantibodies conversion and titer in the context of HLA-DR3 and HLA-DR4 matching. Another important limitation of this study is the missing HLA-DQ typing information. The DR4-DQ8 and DR3-DQ2.5 haplotypes are frequently observed in T1D patients. This is all the more important given that islet-infiltrating human CD4 T cells have also an HLA-DQ restricted repertoire against insulin-derived peptides (37) (38). Fourthly, as only single islet infusions were included in this study, it will be important to examine in future studies the role of HLA-haplotype matching in insulin-independence after multiple islet infusions. Finally, the heterogenous immunosuppression protocols given (including on the use of T cell depleting reagents) may represent a cofounding factor in this study.

In conclusion, our results suggest that matching HLA-DR but excluding the diabetogenic HLA-DR3 and/or 4 is a strong predictor for insulin-independence after single islet infusion. Further studies to confirm and extend the role of diabetogenic HLA DR and associated haplotypes with higher resolution and for multiple islet donors are now needed. Importantly, the feasibility of avoiding the diabetogenic HLA-DR in clinical practice need to be addressed.

## Data availability statement

The raw data that support the findings of this study may be available on request from the Collaborative Islet Transplant Registry Coordinating Center.

## Ethics statement

CITR data are rigorously monitored by the Data Coordinating Center, The EMMES Corporation, Rockville, Maryland, to comply with U.S. Food and Drug Administration Part 21 Code of Federal Regulations requirements. Site participation in the Registry requires local research ethics board approval, strict assurance of patient-identifier confidentiality, and written informed consent by the islet recipients. The CITR Publications and Presentations Committee approved the manuscript.

## Author contributions

CB and FB researched data and performed the analysis. EP researched data and contributed to discussion. YM, JV, RM and TB designed the original project. YM wrote the manuscript. DB, RA, RM, TE and MK contributed to the discussion and reviewed/edited the manuscript. All authors contributed to the article and approved the submitted version.
